# Association between dynamic resting-state functional connectivity and ketamine plasma levels in visual processing networks

**DOI:** 10.1038/s41598-019-46702-x

**Published:** 2019-08-07

**Authors:** Marie Spies, Manfred Klöbl, Anna Höflich, Allan Hummer, Thomas Vanicek, Paul Michenthaler, Georg S. Kranz, Andreas Hahn, Dietmar Winkler, Christian Windischberger, Siegfried Kasper, Rupert Lanzenberger

**Affiliations:** 1 Department of Psychiatry and Psychotherapy, Medical University of Vienna, Vienna, Austria; 2 MR Center of Excellence, Center for Medical Physics and Biomedical Engineering, Medical University of Vienna, Vienna, Austria; 3 Department of Rehabilitation Sciences, The Hong Kong Polytechnic University, Hung Hom, Hong Kong China

**Keywords:** Psychiatric disorders, Magnetic resonance imaging, Experimental models of disease

## Abstract

Numerous studies demonstrate ketamine’s influence on resting-state functional connectivity (rsFC). Seed-based and static rsFC estimation methods may oversimplify FC. These limitations can be addressed with whole-brain, dynamic rsFC estimation methods. We assessed data from 27 healthy subjects who underwent two 3 T resting-state fMRI scans, once under subanesthetic, intravenous esketamine and once under placebo, in a randomized, cross-over manner. We aimed to isolate only highly robust effects of esketamine on dynamic rsFC by using eight complementary methodologies derived from two dynamic rsFC estimation methods, two functionally defined atlases and two statistical measures. All combinations revealed a negative influence of esketamine on dynamic rsFC within the left visual network and inter-hemispherically between visual networks (p < 0.05, corrected), hereby suggesting that esketamine’s influence on dynamic rsFC is highly stable in visual processing networks. Our findings may be reflective of ketamine’s role as a model for psychosis, a disorder associated with alterations to visual processing and impaired inter-hemispheric connectivity. Ketamine is a highly effective antidepressant and studies have shown changes to sensory processing in depression. Dynamic rsFC in sensory processing networks might be a promising target for future investigations of ketamine’s antidepressant properties. Mechanistically, sensitivity of visual networks for esketamine’s effects may result from their high expression of NMDA-receptors.

## Introduction

Over the last two decades, ketamine has developed significant importance in the field of psychiatry as a vehicle for the investigation of glutamatergic mechanisms in psychiatric disorders and their treatment. On the one hand, ketamine evokes profound, reproducible, and rapid antidepressant effects^1^ and is thus utilized as an off-label therapy option for patients with treatment-resistant depressive disorders^2,3^. Ketamine is understood as a glutamatergic antidepressant^4^ and its efficacy is seen as evidence for the glutamatergic theory of depressive pathophysiology^5^. On the other hand, ketamine has been harnessed for investigation of glutamatergic psychosis models^6–11^.

Resting-state functional connectivity (FC) is strongly mediated by the glutamatergic system^12–14^ and is therefore highly reflective of – and an appropriate target for – investigation of ketamine’s effects on the human brain. To date, several functional magnetic resonance imaging (fMRI) studies have examined changes to rsFC during and after ketamine administration. We previously demonstrated increased connectivity between the thalamus and cortical regions during esketamine infusion in healthy controls^15^. Other studies found a ketamine-related increase in rsFC between the hippocampus and prefrontal cortex (PFC)^16^ and from the hippocampus to a broad spectrum of cortical and subcortical regions^17^. Decreased rsFC as a result of ketamine administration has been demonstrated between the hippocampus and other cortical regions^18^ as well as within the default mode network^19^.

The apparent variability in the connectivity patterns reported by individual studies may, to some extent, result from differences in methodology. Seed-based methods require a-priori seed selection^20^. However ketamine likely exerts circuit-specific influence^21^ and seed-based assessment methods only offer snap shots of some of these effects. This point can be addressed through use of whole-brain rsFC estimation methods that do not rely on seed selection.

In fact, the influence of ketamine administration on rsFC has also been assessed with whole-brain approaches^22,23^. For example, a post-ketamine increase in global connectivity within the PFC was demonstrated in patients with major depressive disorder. Global rsFC was defined as the averaged connectivity of a specific voxel to the rest of the brain^22,23^.

Studies reporting on rsFC under or after ketamine administration often treat rsFC patterns in a static manner, i.e. ketamine vs. placebo^9^. However, preclinical evidence suggests that rsFC should rather be understood as a dynamic measure^24^. One study by our group assessed rsFC changes over time, but with coarse temporal resolution^15^. Recently, novel approaches allowing for estimation of dynamic changes to rsFC with higher temporal resolution in humans were developed^24^. Among these are the exponentially weighted sliding-window correlation method^25^ and multiplication of temporal derivatives (MTD)^26^. In general, the atlases used for rsFC estimation are often based on anatomical data and vary between studies, which creates potential for parcellation-specific effects^27,28^. The influence of these methodological differences may be addressed through utilization of multiple rsFC estimation methods and atlases within one data set.

We thus aimed to assess the influence of ketamine on rsFC using whole-brain dynamic rsFC estimation methods. We utilized a set of approaches that were optimized to address methodological shortcomings of current ketamine rsFC studies. Based on the limitations associated with static rsFC assessment^24^, two complementary, dynamic rsFC estimation methods (exponentially weighted sliding-window correlation and MTD) were used. To mitigate parcellation effects, each approach was performed with two whole-brain fMRI-based atlases^29,30^. Regression analysis was used to model the influence of esketamine plasma levels, derived from a pilot study, on rsFC assessed with these dynamic methods. Two statistical measures derived from network based statistics (NBS)^31^ were then used to elucidate networks in which esketamine significantly influenced rsFC. Thus, a total of 8 whole-brain dynamic rsFC estimation methodologies were utilized within the same data set, hereby allowing for elucidation of only highly reproducible, robust changes to dynamic rsFC.

## Results

**Figure 1:**
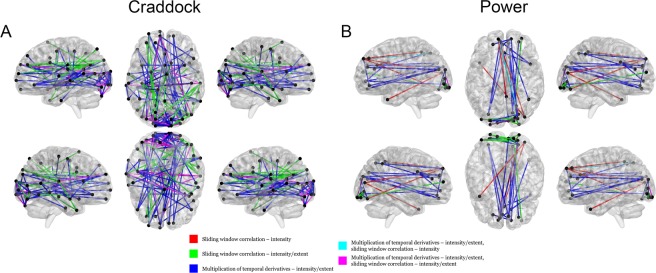
Dynamic rsFC networks in which NBS revealed a significant influence of esketamine. NBS^31^ was performed with each of the 8 methodological combinations to probe for networks in which esketamine had a significant influence on dynamic rsFC in comparison to placebo. Significant networks were only shown for the contrast esketamine < placebo, i.e. esketamine only demonstrated significantly negative effects on dynamic rsFC. Except for three combinations, one significant network per rsFC estimation method, per atlas, per statistical measure (intensity/extent) was revealed. See Table 1 for additional information on the networks elucidated. Red, green, dark blue, light blue and green lines denote with which statistical methods (intensity and/or extent based on NBS^31^) on which rsFC data (exponentially weighted sliding-window correlation method based on Zalesky *et al*.^25^ and/or MTD based on Shine *et al*.^26^) edges (connections) are significant. A and B denote parcellation based on Craddock *et al*.^29^ and Power *et al*.^30^.

**Figure 2:**
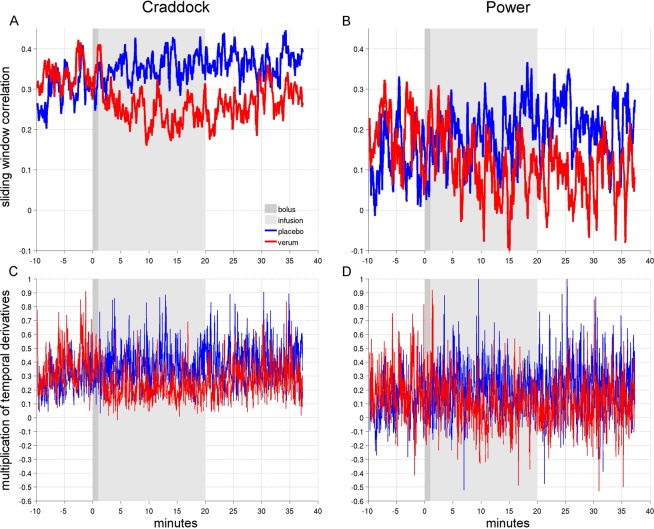
Time-course of average dynamic rsFC under esketamine and placebo. Esketamine reduced dynamic rsFC in comparison to placebo. Figure displays rsFC averaged from connections for which NBS revealed that correlation with esketamine plasma level time-course was shown to be significantly stronger for the test condition than for the placebo condition in all methodological approaches (Table 1). Red and blue lines denote average dynamic rsFC under esketamine and placebo, respectively. Exponentially weighted sliding-window correlation method based on Zalesky *et al*.^25^ (**A,B**) and MTD based on Shine *et al*.^26^ (**C,D**) were utilized for estimation of dynamic rsFC. Both procedures were performed using the atlases by Craddock *et al*.^29^ and Power *et al*.^30^. Grey bars demark esketamine administration (Esketamine i.v. (0.11 mg/kg bolus over one minute (dark grey) followed by 0.12 mg/kg over 19 minutes (light grey))/Placebo i.v. (0.9% NaCl)).

NBS revealed that esketamine had a significant negative effect on dynamic rsFC in comparison to placebo. Figure 1 and Table 1 illustrate networks elucidated through NBS, in which the esketamine and placebo conditions differed significantly (p < 0.05), i.e., the correlation between esketamine plasma level time-course and dynamic rsFC differed between conditions within these networks. Except for three combinations, one significant network per rsFC estimation method, per atlas, per statistical measure (intensity/extent) was revealed. Two networks were found for the exponentially weighted sliding-window method performed with intensity statistics on the Power atlas^30^ as well as for MTD, using both statistics (extent and intensity), when applied to the Craddock parcellation^29^. Each network was found for the comparison esketamine < placebo condition. This means that the influence of esketamine plasma levels on dynamic rsFC time-course within the network, and on each individual connection comprising the network, was negative in comparison to placebo. For individual connections shown by NBS to be robustly significant across all combinations of rsFC estimation methods and statistics see Table 2. For the dynamic rsFC time-course of these overlapping significant connections, see Figure 2.

**Table 1:** Networks in which NBS revealed a negative effect of esketamine on dynamic rsFC.

Atlas	Statistic	p-value	Edges	Nodes
Esketamine < placebo				
**Exponentially weighted sliding-window**				
Craddock	Extent	0.004	45	36
Craddock	Intensity	0.002	45	36
Power	Extent	0.017	14	10
Power	Intensity	0.003	14	10
Power	Intensity	0.023	5	6
**Multiplication of temporal derivatives**				
Craddock	Extent	0.033	25	19
Craddock	Extent	0.005	57	27
Craddock	Intensity	0.024	25	19
Craddock	Intensity	0.003	57	27
Power	Extent	0.005	23	23
Power	Intensity	0.005	23	23

Illustrates networks elucidated through NBS^31^. In these networks, regression coefficients between dynamic rsFC time-course and esketamine plasma levels differed significantly between the esketamine and placebo conditions, demonstrating a significant effect of esketamine on dynamic rsFC. All networks were found for the comparison esketamine < placebo.

Edges = number of connections, nodes = number of regions.

Exponentially weighted sliding-window based on Zalesky *et al*.^25^, multiplication of temporal derivatives based on Shine *et al*.^26^.

Craddock and Power denote parcellations based on Craddock *et al*.^29^ and Power *et al*.^30^.

Extent and intensity statistics based on NBS^31^.

**Table 2:** Individual connections for which NBS revealed a negative influence of esketamine on dynamic rsFC across all methodological approaches.

Craddock atlas				
Coordinates		Regions (AAL)		Network
−68/−28/0	64/−24/16	Mid. temporal gyrus (l)	Sup. temporal gyrus (r)	lDM-rSM
64/−24/16	−48/−32/0	Sup. temporal gyrus (r)	Mid. temporal gyrus (l)	rSM-lDM
20/−76/−4	−32/−88/12	Lingual gyrus (r)	Mid. occipital gyrus (l)	rVI-lVI
−32/−88/12	−8/60/28	Mid. occipital gyrus (l)	Sup. med. frontal gyrus (l)	lVI-lDM
20/−76/−4	28/−96/0	Lingual gyrus (r)	Inf. occipital cortex (r)	rVI-rVI
−20/−96/−8	−20/−72/−8	Inf. occipital cortex (l)	Lingual gyrus (l)	lVI-lVI
20/−76/−4	−20/−72/−8	Lingual gyrus (r)	Lingual gyrus (l)	rVI-lVI
28/−96/0	−20/−72/−8	Inf. occipital cortex (r)	Lingual gyrus (l)	rVI-lVI
8/−100/−8	−20/−72/−8	x	Lingual gyrus (l)	rVI-lVI
−48/−32/0	64/−24/−12	Mid. temporal gyrus (l)	Mid. temporal gyrus (r)	lDM-rDM
−16/−64/28	0/0/−8	Sup. occipital region (l)	x	lVI-rBG
64/−24/16	0/0/−8	Sup. temporal gyrus (r)	x	rSM-rBG
20/−76/−4	−40/−84/−12	Lingual gyrus (r)	Inf. occipital cortex (l)	rVI-lVI
−68/−28/0	44/12/−16	Mid. temporal gyrus (l)	Sup. temporal pole (r)	lDM-rDM
−8/60/28	48/−80/8	Sup. med. frontal gyrus (l)	Mid. occipital gyrus (r)	lDM-rVI
−32/−88/12	12/−100/16	Mid. occipital gyrus (l)	x	lVI-rVI
28/−96/0	12/−100/16	Inf. occipital cortex (r)	x	rVI-rVI
−40/−84/−12	12/−100/16	Inf. occipital cortex (l)	x	lVI-rVI
20/−76/−4	−8/−84/−28	Lingual gyrus (r)	Crus 2 cerebellum (l)	rVI-lCE
8/−100/−8	−8/−84/−28	x	Crus 2 cerebellum (l)	rVI-lCE
**Power atlas**				
**Coordinates**		**Regions (AAL)**		**Network**
−24.66/−97.84/−12.33	24.41/−87.21/24.01	x	Sup. occipital region (r)	lVI-rVI
7.98/−91.08/−7.10	−40.21/−88.44/−6.19	Lingual gyrus (r)	Mid. occipital gyrus (l)	rVI-lVI

Illustrates individual region to region connections for which NBS^31^ showed that esketamine had a significant negative influence on dynamic rsFC regardless of which methodological approach was used. Coordinates from the Craddock and Power atlases were translated to AAL and corresponding Yeo networks are noted to improve interpretability.

AAL = Automated Anatomical Labeling atlas.

x = coordinates from Craddock atlas^29^ or Power atlas^30^, but not denoted in AAL.

rVI/lVI: right/left visual network,*

rSM/lSM: right/left somato-motor network,*

rDM/lDM: right/left default mode network,*

rBG/lBG: right/left basal ganglia,**

rCE/lCE: right/left cerebellum**.

*Networks based on Yeo *et al*.^32^, **Regions based on Harvard-Oxford atlas.

Carry-over effects, i.e. effects of the sequence in which subjects received study drug, were probed for using NBS. No carry-over effects were detected in any of the esketamine-sensitive networks.

**Figure 3:**
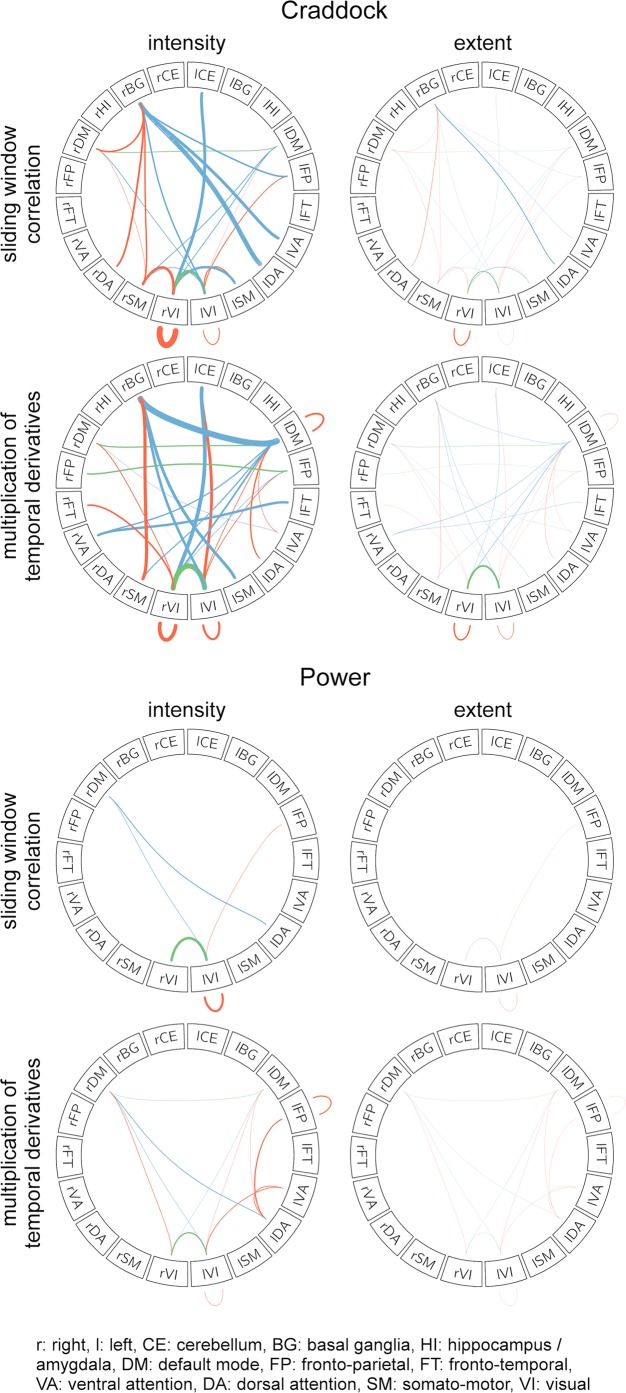
Esketamine-sensitive within- and inter-network dynamic rsFC connections, revealed after compartmentalization into Yeo networks. Connections for which NBS^31^ showed a negative influence of esketamine on dynamic rsFC were compartmentalized into networks based on Yeo *et al*.^32^ and three additional regions (amygdala/hippocampus, basal ganglia, cerebellum, based on the Harvard-Oxford atlas). This figure depicts connections within and between these networks/regions (Yeo/Harvard-Oxford), split for each methodological approach (exponentially weighted sliding-window correlation method based on Zalesky *et al*.^25^, MTD based on Shine *et al*.^26^), atlas (Craddock *et al*.^29^, Power *et al*.^30^), and statistic (intensity: strength of connections, extent: number of connections). Esketamine plasma levels had a significant negative influence on dynamic rsFC in comparison to placebo for the connections between LVI and RVI and within the LVI, regardless of dynamic rsFC estimation method. For a list of connections see Table S1. Orange, green, and blue lines reflect connections within networks of the same hemisphere, inter-hemispheric connections within the same network, and inter-hemispheric connections to another network, respectively. Line thickness reflects intensity/extent statistics, depending on the subfigure. rVI/lVI: right/left visual network,* rSM/lSM: right/left somato-motor network,* rDA/lDA: right/left dorsal attention network,* rVA/lVA: right/left ventral attention network,* rFT/lFT: right/left fronto-temporal network,* rFP/lFP: right/left fronto-parietal network,* rDM/lDM: right/left default mode network,* rHI/lHI: right/left amygdala/hippocampus** (not included in the Power parcellation^30^), rBG/lBG: right/left basal ganglia,** rCE/lCE: right/left cerebellum**. *Networks based on Yeo *et al*.^32^, **Regions based on Harvard-Oxford atlas.

NBS results were then compartmentalized into networks based on Yeo *et al*.^32^ in order to improve their interpretability. Esketamine showed a significant negative influence on dynamic rsFC within the left visual network (LVI) and on inter-hemispheric dynamic rsFC between the LVI and right visual network (RVI). Though other connections could be replicated utilizing either both rsFC estimation methods or statistical measures, the negative influence on dynamic rsFC within the LVI and on inter-hemispheric dynamic rsFC between the LVI and RVI was consistent for all 8 methods, see Figure 3 and Table S1.

Furthermore, post-hoc correlation analysis was performed between the influence of esketamine on dynamic rsFC and the visionary restructuralization (VRS) sub-scale of the 5-Dimensional Altered States of Consciousness Rating Scale (5D-ASC) by Dittrich *et al*.^33^, as well as the individual questions comprising this sub-scale. These variables were chosen because dynamic rsFC was found to be sensitive to esketamine in visual processing networks. This analysis revealed a positive Spearman correlation with “shapes seemed to be changed by sounds or noises” (*ρ* = 0.401, MTD, Power atlas), “some everyday things acquired special meaning” (*ρ* = 0.488, MTD, Craddock atlas; *ρ* = 0.381, MTD, Power atlas) and “I saw brightness or flashes of light with closed eyes or in complete darkness” (*ρ* = 0.425, exponentially weighted sliding-window, Craddock atlas), all p < 0.05 uncorrected.

Age did not differ significantly between the pilot (mean age ± SD = 25.40 ± 2.80, 3 males) and main study (mean age ± SD = 24.52 ± 4.60, 13 males) groups.

## Discussion

We assessed the influence of ketamine on whole-brain, dynamic fMRI rsFC using 8 complementary methodological combinations. Thus, any findings that were replicated with all approaches can be considered highly robust effects of esketamine administration on dynamic rsFC. In a first step, whole-brain NBS assessment isolated networks in which the association between the time-course of dynamic rsFC and esketamine plasma levels differed significantly between the esketamine and the placebo conditions. Significant networks were only isolated for the comparison esketamine < placebo, i.e., esketamine had an exclusively negative influence on dynamic rsFC. After compartmentalization of NBS results into the functional networks described by Yeo *et al*.^32^, all combinations of methods revealed a reduction of dynamic rsFC by esketamine within the LVI and between the LVI and RVI.

Numerous fMRI studies have investigated ketamine’s effects on rsFC. However, the reported results vary widely. In addition, many studies only address rsFC within or between specific regions or networks to begin with. In healthy controls, network-specific increases^15–17^ and decreases^18,19^ have been described. Some studies report changes in both directions, depending on the networks investigated^34^. These studies underline the importance of utilizing whole-brain rsFC approaches because they demonstrate that ketamine induced changes to rsFC cannot be generalized across the brain. Non whole-brain rsFC analyses utilizing singular or few seeds can only offer localized assessments of ketamine’s effects^20^, which are likely regionally different and network-specific^21^. In fact, the divergence between results from our previous rsFC study published on a largely overlapping resting-state data set^15^ and the current results can be linked to methodological differences. In comparison to the dynamic, whole brain approach used in the current study, the previous study focussed on the thalamo-cortical network utilizing a static, seed-based approach^15^. Therefore, the networks elucidated through whole-brain NBS in the study at hand could not be assessed.

In our whole-brain fMRI study, we assess dynamic rsFC, which provides information on the time-course of ketamine’s effects on rsFC. The dynamic nature of FC is made apparent by reactivity of the measure to task environments^24,25^. Fluctuations in FC occur within very short time-frames, an obvious characteristic when considering that switching between cognitive states, which can occur rapidly, is accompanied by changes to FC. This phenomenon is demonstrated by a whole-brain FC study in healthy controls which showed that the brain fluctuates between cognitive states, which can be detected over fMRI time-frames as short as 30–60 seconds, and that these undulations are echoed by fluctuations in FC^35^.

To date, most studies investigating ketamine’s effects on rsFC in humans have utilized static assessment approaches. In fact, recent assessments of ketamine’s effects on static rsFC contradict our results by suggesting that, while default mode network and salience network connectivity is reduced, ketamine does not affect rsFC in visual processing networks^36^. However, various methodological differences, including the use of static rsFC assessment methods, different ketamine dosages, as well as diverging administration times, might be responsible for these differences^36^. A recent study also utilized dynamic rsFC assessment approaches to investigate ketamine’s effects in humans, though the methods used differ substantially from those used in our study. Joules *et al*. used a windowed-approach and graph theory to demonstrate that ketamine results in a shift from cortical to subcortical connectivity^37^. While we implemented a frame-length of one minute, the previous study used a substantially longer window-length of four minutes. In addition, Joules *et al*. base their parcellation on the Automated Anatomical Labeling (AAL) atlas^37^, which was shown to exhibit low functional homogeneity, which may bias results^38,39^. Furthermore, the previous study used one parcellation. However, as illustrated by Figure 3 and Table S1, choice of parcellation and estimation methodology influence results. Through utilization of various approaches for estimation of dynamic rsFC, our study allows for elucidation of robust findings. Our results suggest that esketamine’s influence on dynamic rsFC might be most stable in visual processing networks. Visual processing networks may express a particular propensity for ketamine sensitivity due to their high expression of NMDA-receptors, as demonstrated by post-mortem studies^40^.

Ketamine is utilized as a model for the glutamatergic theory of psychosis^6–10^. Information on the time-course of ketamine’s effects on rsFC has implications for our understanding of ketamine as a model for this disease. If ketamine’s effects are to be interpreted as a psychosis-model, it would be an oversimplification to define “ketamine” and “non-ketamine” rsFC effects, as this would be paramount to defining binary “psychotic” and “non-psychotic” states. However, static rsFC methods do just this^25^. Dynamic rsFC assessments and regression with esketamine plasma levels allows for more specific assessment of the influence of ketamine on rsFC. Our results are in line with previous studies showing that ketamine exerts influence on visual processing regions and networks, and that these changes might be understood as part of the substance’s role as a psychosis model. Animal studies have demonstrated that ketamine induces phase-coupling of gamma oscillations within the visual cortex. This is thought to be a fundament for aberrant processing of bottom-up information, and might be interpreted as a correlate for visual hallucinations^41^, which ketamine is known to elicit^42^. In addition to decreasing dynamic rsFC within the LVI, a reduction in inter-hemispheric connectivity between the LVI and RVI under ketamine was found. Several studies show impaired inter-hemispheric rsFC in schizophrenia^43–45^. In the context of ketamine’s utilization as a model for psychosis, our results suggest that ketamine might reflect alterations to inter-hemispheric FC as found in schizophrenia.

Studies utilizing dynamic rsFC have also repeatedly demonstrated various changes to sensory processing networks in patients with schizophrenia. Altered connectivity between visual, auditory, sensorimotor, and subcortical regions was shown^46^. In addition, dynamic rsFC studies in schizophrenia showed increased temporal^47^ and regional variability^48^ in visual processing networks. Increased variance of connectivity was also detected in schizophrenia within the salience network^49^. Therefore, our dynamic rsFC findings under esketamine might mirror dynamic rsFC findings in schizophrenia that show altered connectivity in sensory processing networks^46^, more specifically in visual processing networks^48^. Our results therefore further corroborate the concept of utilizing ketamine as a model for psychosis^6–10^.

On the other hand, the results of our post-hoc correlation analysis did not reveal an association with VRS derived from the 5D-ASC. We found that the influence of esketamine on dynamic rsFC correlated with specific 5D-ASC^33^ items. Positive correlations were found with “some everyday things acquired special meaning,” “shapes seemed to be changed by sounds or noises,” and “I saw brightness or flashes of light with closed eyes or in complete darkness,” though these effects did not survive correction for multiple comparisons. Considering that esketamine had a negative influence on dynamic rsFC in the visual processing regions included in this post-hoc correlation analysis, a positive correlation would indicate that the lower the influence of esketamine, the stronger these clinical effects were. Interestingly, though not statistically significant, a negative relationship was found with the item “I saw things I knew were not real,” which might more directly reflect visual hallucinations. On the one hand, a lack of a correlation might be seen as contradictory to the idea that dynamic rsFC changes under ketamine are reflective of those during psychosis. However, the lack of a significant correlation with VRS in general and hallucinations in particular might be based on inconsistency in how subjects interpreted their findings and thus reported, i.e. how they translated their experiences to the questionnaire. This is reflected by the broad variability in this subject group’s VRS scores (mean VRS ± SD = 44.27 ± 35.67), which represents a general limitation of self-rating scores.

Ketamine was shown to induce nystagmus in healthy individuals. It also elicits other oculomotor abnormalities including changes to smooth pursuit eye movement that are independent of nystagmus^50^. The NMDA receptor is likely implicated in the oculomotor changes ketamine induces^51^. Investigations into rsFC during nystagmus are limited. However, optokinetic nystagmus was shown to affect resting-state activity in cortical eye fields^52^. Interestingly, alterations to smooth pursuit movements have also been shown in schizophrenia^53^ and in patients at a genetic risk for schizophrenia^54^. Oculomotor abnormalities may even be a measure of neuropsychological function in the disorder^55^. Though eye movement was not assessed in this study, oculomotor changes elicited by esketamine might theoretically serve as a correlate for the dynamic rsFC changes we detected in visual processing networks.

Ketamine’s effects on visual processing networks might also cautiously be interpreted with ketamine’s antidepressant effects in mind^1^. Depression has been discussed as a visual perceptive disorder. This concept is based on findings demonstrating innervation of visual processing regions by serotonin and noradrenalin^56^ together with strong evidence that depression^57–59^ and antidepressant treatment^59–61^ are associated with alterations to brain activation patterns during processing of emotional faces. In addition, the behavioural correlates of visual processing are altered in patients with depression^62^. Furthermore, studies assessing static and dynamic rsFC have shown alterations in FC between the salience network and other brain networks in depressed patients^63^, potentially highlighting more broad changes to processing of sensory input in depression. Together with our results demonstrating esketamine-related alterations to dynamic rsFC in visual processing networks, these studies suggest that changes to sensory processing might be promising targets for future investigations into correlates for ketamine’s antidepressant properties.

However, interpretation of our results in the context of ketamine’s antidepressant effects^1^ must be understood as purely hypothetical because we performed our study in healthy subjects. Depression is associated with alterations to the glutamatergic system^5,22^. As we assume that our healthy controls do not show baseline glutamatergic pathology, it cannot simply be presumed that the esketamine-related alterations to dynamic rsFC in our subject group necessarily overlap with those in depressed subjects. Furthermore, ketamine’s effects on neural plasticity are proposed to contribute to its antidepressant effects^5^. Ketamine elicits rapid effects on neuroplasticity^64^, however it is nevertheless unlikely that these fall within the time-frame of our measurement. It was shown that ketamine results in activation of the mTOR pathway and production of proteins involved in synaptogenesis after 1 hour^64^. These results suggest that the effects we measure reflect ketamine’s effects on circuitry, rather than on neuroplasticity, and make a direct link between the dynamic rsFC effects we detected and ketamine’s antidepressant properties unlikely. However, the presented evidence proposes that future studies should investigate the broader relevance of alterations to sensory processing to ketamine’s antidepressant effects.

Nevertheless, several limitations must be discussed. We utilized esketamine plasma levels drawn from a pilot sample that did not undergo MRI measurement. This may incorporate variability, particularly as ketamine metabolism and plasma levels have been shown to differ between individuals^65,66^. However, blood draw during MRI would not be compatible with resting-state measurement. Therefore, utilization of pilot esketamine data, as also performed in our previous study, can be seen as an acceptable compromise that allows for assessment between dynamic rsFC and plasma level time-course^15^. Furthermore, the demographic variables of both groups were comparable. We refrained from incorporation of demographic variables into the esketamine plasma level models as this would have resulted in overfitting. In addition, the resting-state fMRI run length is comparably long^67^. The long acquisition time utilized in this study carries the benefit of allowing for assessment of ketamine associated changes to dynamic rsFC over time, but conversely may increase propensity for motion artifacts^68^. In fact, motion increased over the time of the measurement, as to be expected. However, motion artefacts were corrected for through removal of artifact-distorted time-frames and those corresponding to severe motion.

It should be mentioned that which motion correction methodology is most appropriate for dynamic rsFC analyses is a matter of debate. We utilized a data scrubbing approach based on Power *et al*.^69^. It has been suggested that censoring frames, as this method does, may disrupt the time-course of the data. This could in theory affect results if the methodology used for connectivity analysis takes temporal structure into account. However, in the case of our manuscript, this argument is only relevant for the exponentially weighted sliding-window correlation^25^. The MTD approach does not calculate local connectivity estimates if at least one frame is missing. In MTD, all subsequent analyses (i.e., regression and NBS inferences) are agnostic towards the underlying temporal structure^26^. In fact, these mathematical differences underline the importance of our multi-methodological approach. Regarding dynamic rsFC in general, other authors have reported that a potential effect on the data time-course would only be relevant to transient connectivity states that were motion-related^69^, which are of no interest in the current analysis. Furthermore, while deleting single points from a correlation based on little data might lead to more extreme coefficients, replacement by interpolation would not provide additional information but introduces further temporal dependencies if data outside of the current window is used. In our case, assuming that no true effect is present, any residual influence of the missing data points should cancel out at the group level.

Long acquisition times may also increase the propensity for falling asleep. In addition to the influence on vigilance, this might affect rsFC because differences in resting-state fMRI have been shown depending on whether subjects keep their eyes open or closed^70^. In our study, subjects were instructed to keep their eyes open during resting-state fMRI and to fixate on a white cross in order to reduce the risk of falling asleep. In fact, the eyes-open condition was previously associated with higher test-retest reliability^71^, even in regards to rsFC within the visual network^72^. Nevertheless, despite these methodological strengths, in absence of the availability of eye tracking data we cannot exclude the possibility that an influence of ketamine on vigilance resulted in subjects closing their eyes, with potential consequences for dynamic rsFC.

This study, which was placebo-controlled and cross-over, aimed for a double-blind design. However, one of the major limitations of ketamine neuroimaging research in general is the lack of an optimal placebo control. In clinical studies benzodiazepines such as midazolam may be used as an active control. However, even use of benzodiazepines has limitations as these are mostly sedative and ketamine also elicits psychotomimetic effects, which may also result in unblinding^73^. Furthermore, control with an active comparator is problematic in a pharmaco-fMRI study in that it only allows for assessment of the neurobiological differences between the two active substances, and not the effects of ketamine in general. Therefore, use of 0.9% saline as a placebo, as is often used in ketamine rsFC studies^16,17,19,37^, was also deemed the most appropriate option in this study.

Another constraint exhibited by dynamic rsFC assessment methods is that direct comparability with static rsFC estimation methods is not straightforward. We assessed rsFC over a time-period of 60 minutes during which ketamine plasma levels and rsFC change^24^. A statistical comparison with a conventional static rsFC method could only be performed with rsFC averaged over longer time-periods or with arbitrarily chosen time-points or time-frames. The first approach would not allow for regression analysis with esketamine plasma levels, resulting in a lack of information regarding the causality of the relationship between esketamine and rsFC. Furthermore, the meaningfulness of a direct comparison of dynamically assessed rsFC and rsFC averaged over a long time-period during which esketamine plasma levels vary, is questionable. Though the second approach might allow for regression analysis, the time-points and time-frames selected for static assessment could only be chosen arbitrarily. In addition, if multiple time-points or time-frames are chosen, the data attained is in fact dynamically assessed rsFC, however with much lower temporal resolution than with the methods used in our study (exponentially weighted sliding-window^25^ and MTD)^26^. The resulting interpretability of a subsequent statistical comparison to rsFC data assessed dynamically is therefore limited.

In summary, we use 8 methodological combinations derived from two complementary dynamic rsFC estimation methods (exponentially weighted sliding-window^25^ and MTD^26^), two functional atlases (Craddock *et al*.^29^ and Power *et al*.^30^), and two statistical approaches (extent and intensity, as defined by NBS^31^) to isolate robust effects of esketamine administration on dynamic rsFC. NBS and compartmentalization of significant results into functional networks revealed that esketamine had a negative effect on dynamic rsFC within the LVI and between the LVI and RVI. Our results suggest that esketamine’s influence on dynamic rsFC may be most stable in visual processing networks. Visual processing networks may express a particular sensitivity to esketamine’s effects due to their high expression of NMDA-receptors^40^. A negative influence of esketamine on dynamic rsFC within visual processing networks is in agreement with ketamine’s role as a model for psychosis^42^. In addition, a reduction of inter-hemispheric dynamic rsFC by esketamine is in accordance with studies demonstrating impaired inter-hemispheric rsFC in schizophrenia^43–45^. Considering that ketamine elicits profound antidepressant effects in clinical studies^1^ and that depression is associated with changes to sensory processing^56^, our results propose that dynamic rsFC in visual processing networks may be a promising target for future investigations into the neurobiological correlates of ketamine’s antidepressant effects.

## Materials and Methods

### Study design

This work utilizes rsfMRI data from a study previously published by our group^15^ and performed at the Medical University of Vienna, Austria. The study was approved by the Ethics Committee of the Medical University of Vienna and performed according to the Declaration of Helsinki. In this study, 30 healthy subjects were randomized to one of two cross-over treatment arms and were measured twice with MRI. Study physicians and subjects were blinded to which of the substances were applied in each measurement. Subjects randomized to the first treatment arm received esketamine during a first MRI (MRI1) followed by placebo control during a second MRI (MRI2). The other treatment arm consisted of the same study drug (esketamine) and placebo, yet administered in the opposite sequence (placebo during MRI1 and esketamine during MRI2).

### Subjects

fMRI data from 27 of these 30 healthy subjects (mean age ± SD = 24.52 ± 4.60, 13 males) were included in the current analyses. Data from three subjects were removed due to excessive motion artefacts (see “fMRI data preprocessing” for details). Subjects were recruited via information flyers posted at the Medical University of Vienna. As described in Höflich *et al*.^15^, a thorough screening procedure was performed. The Structured Clinical Interview for DSM-IV was administered in order to exclude any Axis-I psychiatric disorders. A medical history and physical examination including electrocardiography and blood draw were performed to exclude severe internal or neurological disorders. Pregnant, tested for using urine-testing, and currently breastfeeding females were excluded from study participation. Urine poly-drug testing was utilized to exclude current drug use. In addition, any former substance-abuse or -dependency, current medication intake, intake of psychopharmaceuticals within the last 6 months, and lifetime intake of antipsychotic drugs were considered exclusion criteria. Furthermore, subjects were excluded if they had any MRI contraindications such as non-MRI compatible implants, pacemakers, or severe claustrophobia. All participants provided written informed consent and received financial reimbursement for participation.

### MRI data acquisition

RsFC measurements were performed using a 3 Tesla Siemens Trio (Erlangen, Germany) scanner installed at the MR Center of Excellence, Center for Medical Physics and Biomedical Engineering, at the Medical University of Vienna. In this 27-subject analysis, measurements were 11.93 ± 5.62 days (mean ± SD) apart. As previously described in Höflich *et al*.^15^, a single-shot gradient-recalled echo-planar imaging sequence (TR/TE: 1.8/0.038 sec, matrix: 128 × 128 voxel, field of view: 190 × 190 mm, 23 slices, resulting in a voxel size of 1.48 × 1.48 × 3.0 mm and a slice gap of 1.8 mm) was used. Subjects were instructed to keep their eyes open, focus their view on a white cross on a dark background, and to relax without falling asleep. Per protocol, resting-state scans were acquired over the course of 60 minutes, though scans were terminated early in some cases, due to subject request or technical issues, resulting in acquisition of a median of 1913.5 frames (maximum/minimum: 1943/1745 frames).

### Esketamine administration and MRI protocol

During each MRI measurement, subjects received either esketamine or placebo. In this study, esketamine was used because ketamine in the form of the entire racemat was not marketed in Austria at the time of this study. Study drug and placebo were provided in randomized and blinded form by the hospital pharmacy at the Medical University of Vienna. Ketanest S 5 mg/mL ampoules (Actavis Italy S.P.A./Pfizer) were utilized for study drug preparation which included dilution in 0.9% NaCl. Esketamine and placebo were each administered as an intravenous infusion using an MR compatible infusion pump. Administration of study drug or placebo began after 10 minutes of baseline rsFC measurement. 0.9% NaCl was administered with increasing speed for the latter 5 of these 10 baseline rsFC measurement minutes at both time-points in order to reduce the probability that the measurement would be biased in that subjects might recognize the start of esketamine/placebo infusion. Placebo also consisted of 0.9% NaCl administered intravenously. Esketamine was administered as a bolus (0.11 mg/kg) over one minute followed by a continuous infusion (0.12 mg/kg) over 19 minutes. For additional information regarding the esketamine administration scheme see previous publications by Höflich *et al*.^15,74,75^.

### fMRI data preprocessing

Preprocessing including correction for slice-timing effects and head motion artefacts as well as normalization to Montreal Neurological Institute (MNI) space was performed according to Höflich *et al*.^15^ utilizing SPM8 (Wellcome Trust Centre for Neuroimaging, http://www.fil.ion.ucl.ac.uk/spm). All further processing steps were performed in Matlab (version R2014a, https://de.mathworks.com/products/matlab) using in-house code. Detection of outlier frames due to motion artefacts was performed with the ArtRepair toolbox (Version 5b3, Center for Interdisciplinary Brain Sciences Research, Stanford Medicine, http://cibsr.stanford.edu/tools/human-brain-project/artrepair-software.html)^76^. In detail, a modified version of the art_global function was used, which additionally detrends the mean time-course before identifying outliers to avoid signal drifts over the long measurements being misclassified as subject movement. Motion artefacts were further corrected for using the Friston-24 model (6 realignment parameters, realignment parameters with lag 1 and the squared versions of both)^77^. The potential effects of physiologic factors such as respiration and cardiac activity were taken into account by extracting the first 5 principal components of white matter (WM) and cerebrospinal fluid (CSF) regions^78,79^ defined by thresholding the respective maps of the Harvard-Oxford atlas at 95%^80^. As suggested by Hallquist *et al*.^81^, bandpass filtering from 0.01 to 0.1 Hz was performed using sine and cosine regressors. The overall nuisance regression model comprised 24 motion parameters, 5 WM and 5 CSF components, the frequency regressors (exact number depending on the number of available frames for each subject) and the intercept. Frames identified as motion outliers were not included in the regression (minimum/median/maximum: 4/92/518 discarded frames; 0.21%/4.81%/27.08% of frames for respective subject) and – if possible – interpolated during connectivity estimation. Three of the originally randomized 30 subjects for whom this procedure led to underdetermined models (too many censored time-points) were excluded from the analysis, resulting in n = 27. Motion correction for dynamic FC analysis using data scrubbing as described above was previously shown to reduce the influence of in-scanner movement without disturbing integrity of data^69^. Removal of artifact distorted time-frames was not considered to have a substantial effect on results due to the high number of time-points (a median of 1913 frames was recorded) and comparably low number of frames censored (a median of 4.81%).

### Resting-state functional connectivity assessment

Estimation of dynamic rsFC was performed using the exponentially weighted sliding-window correlation method based on Zalesky *et al*.^25^ and MTD based on Shine *et al*.^26^, again using in-house Matlab code. In the first approach based on Zalesky *et al*. an exponentially tapered sliding-window of 60 seconds with a theta parameter of 7, resulting in a minimum weight of 1% compared to the maximum weight, was shifted by 1 TR^82^. With this method rsFC connectivity between each region pair is expressed as an exponentially weighted Pearson correlation for each overlapping 60 second window. If a window included censored frames, the weights of the remaining ones were adapted to account for the missing data (to keep a weight sum of 1). In MTD, temporal derivatives are calculated for each region pair as a backward difference, scaled by their standard deviations and multiplied for each connectivity pair. This method provides TR resolved approximation of the correlation of the time-course between two regions^26^. However, a single censored frame leads to 2 missing MTD time-points as interpolation is not possible. These dynamic rsFC estimation methods were selected because they are complementary, in particular as MTD is not susceptible to the influence of window length^26^. Both methods were performed using the Craddock and Power atlases in order to reduce the chance of parcellation-specific results. The Craddock atlas is based on resting-state fMRI data^29^ while the Power atlas utilizes resting-state and task-based fMRI data^30^. Two atlases were used as previous studies have shown an influence of parcellation approach on rsFC results^28^. Functionally defined atlases were chosen as anatomical parcellations were shown to distort FC results due to the fact that they average signal across ROIs^27^.

### Esketamine plasma levels

In order to relate potential changes in dynamic rsFC to esketamine administration, esketamine plasma levels from a previously published pilot study performed in a different group of 5 healthy controls were utilized (mean age ± SD = 25.40 ± 2.80, 3 males)^75^. These subjects received esketamine in the same administration scheme as participants of the main study, however, no MRI measurements were performed. Eight blood draws were performed up to 140 minutes after the start of esketamine infusion as described in the previous publication by our group^75^. Esketamine plasma levels were determined using high-pressure liquid chromatography at the Experimental Psychiatry Unit, Center of Psychiatry and Psychotherapy, Medical University of Innsbruck, Austria. Quantification of the esketamine curves was performed by averaging esketamine plasma levels across the pilot subjects at each time-point and fitting these values with a power function damped model as performed in Höflich *et al*.^75^.

### Clinical scores

As reported by Höflich *et al*.^15^, the 94 point 5D-ASC developed by Dittrich *et al*.^33^ was performed after each of the measurements. Subjects were instructed to report on the experience they had during the directly preceding infusion and MRI measurement.

### Statistical analyses

The association between dynamic rsFC and esketamine plasma level time-course was computed via ordinary least-squares regression using Matlab. For placebo and verum measurements, the same model curve was used based on the null hypotheses that there is no difference in temporal correlation between both conditions. This procedure was repeated for both rsFC estimation methods (exponentially weighted sliding-window^25^, MTD^26^) and both atlases^29,30^. The resulting matrices of regression coefficients (one coefficient per region-region connection; intercept was included but not further used) were entered into a repeated-measures model and significance was assessed utilizing NBS^31^ with 10,000 permutations. The threshold was chosen as the t-value for p = 0.001 uncorrected at the available degrees of freedom and the significance threshold was set to $\alpha =0.05$. All together, this procedure was repeated for all four approaches (2 estimation methods, 2 atlases) and extent (number of edges (connections) between networks) and intensity (strength of connections between networks) statistics as defined by NBS. In a nutshell, NBS randomizes the labels of data matrices with respect to within-factors and computes t-statistics for each element. The intensity or extent of the largest continuous network is stored for each permutation and used as test metric. A network is thereby defined as set of continuously connected nodes (regions) where the weight of each edge (region-region connection) needs to be above the pre-defined threshold. This way, the multiple testing of matrix elements is reduced to a non-parametric test of the network size/strength. Correction for multiple testing is inherently included in NBS^31^.

In addition, NBS was used to probe for carry-over effects by testing for interaction effects between condition (esketamine, placebo) and measurement (MRI1, MRI2). Differences in age between the pilot and main study groups were tested for using t-test.

In order to improve interpretability of results, significant networks based on NBS were then compartmentalized into networks described by Yeo *et al*. (visual network, somato-motor network, dorsal attention network, ventral attention network, fronto-temporal network, fronto-parietal network, default mode network)^32^, and three additional regions (amygdala/hippocampus, basal ganglia, cerebellum) based on the Harvard-Oxford atlas, each split by hemisphere (right and left for each network/region)^83^. The Yeo atlas was not initially utilized as parcellation for rsFC estimation because it shows poorer homogeneity of signals due to larger ROIs^38^ than the Craddock^29^ and Power^30^ atlases. Compartmentalization into the Yeo atlas allows for description of connections in terms of intensity and extent statistics within networks, between networks within the same hemisphere, and between inter-hemispheric networks. If multiple networks were detected within the same NBS test, they were merged into one.

Furthermore, post-hoc Spearman correlation analyses were performed between the influence of esketamine on dynamic rsFC in the esketamine sensitive networks (mean difference in regression coefficients between the esketamine and the placebo conditions, coefficients refer to regression between dynamic rsFC and the temporal esketamine model) and 5D-ASC based on Dittrich *et al*.^33^. This step was taken in order to relate esketamine’s influence on dynamic rsFC to clinical effects. Based on the finding that esketamine had a significant negative influence on dynamic rsFC in visual processing networks, correlation analysis was performed with the VRS sub-score and the individual 5D-ASC points comprising the VRS sub-score (18 points). Correction was performed using permutation testing (1000 permutations per ROI set).

## Supplementary information

Table S1
