# Supplementary material for: Association between dynamic resting-state functional connectivity and ketamine plasma levels in visual processing networks
Source: Sci Rep. 2019 Aug 7;9:11484. doi: 10.1038/s41598-019-46702-x (PMC6685940; doi:10.1038/s41598-019-46702-x)
Supplement: Supplementary file 1 — Table S1 [file 41598_2019_46702_MOESM1_ESM.docx]

Supplementary Information

Association between dynamic resting-state functional connectivity and ketamine plasma levels in visual processing networks

Marie Spies^a^, Manfred Klöbl^a^, Anna Höflich^a^, Allan Hummer^b^, Thomas Vanicek^a^,

Paul Michenthaler^a^, Georg Kranz^a,c^, Andreas Hahn^a^, Dietmar Winkler^a^,

Christian Windischberger^b^, Siegfried Kasper^a^, Rupert Lanzenberger^a^

*^a^Department of Psychiatry and Psychotherapy, Medical University of Vienna, Austria*

*^b^MR Center of Excellence, Center for Medical Physics and Biomedical Engineering,*

*Medical University of Vienna, Austria*

*^c^Department of Rehabilitation Sciences, The Hong Kong Polytechnic University, Hung Hom, Hong Kong, China*

**Table S1: Within- and inter-network connections for which esketamine had a negative influence on dynamic rsFC, revealed after compartmentalization into networks based on Yeo et al.**

| **Connection** | **Exponentially weighted sliding-window** | | | | **Multiplication of temporal derivatives** | | | |
| --- | --- | --- | --- | --- | --- | --- | --- | --- |
|  | **Craddock atlas** | | **Power atlas** | | **Craddock atlas** | | **Power atlas** | |
|  | **Intensity** | **Extent** | **Intensity** | **Extent** | **Intensity** | **Extent** | **Intensity** | **Extent** |
| Within lVI | X | X | X | X | X | X | X | X |
| Within rVI | X | X |  |  | X | X |  |  |
| lCE-rVI | X | X |  |  | X | X |  |  |
| lDM-lVI | X | X |  |  | X | X | X | X |
| lDM-rVI | X | X |  |  | X | X | X | X |
| lDM-rSM | X | X |  |  | X | X |  |  |
| lDM-rDM | X | X |  |  | X | X | X | X |
| lFP-lVI | X | X | X | X | X | X |  |  |
| lFP-rBG | X | X |  |  |  |  |  |  |
| lVA-rBG | X | X |  |  |  |  |  |  |
| lDA-rBG | X | X |  |  |  |  |  |  |
| lSM-lVI | X | X |  |  |  |  |  |  |
| lSM-rVI | X | X |  |  |  |  |  |  |
| lVI-rVI | X | X | X | X | X | X | X | X |
| lVI-rSM | X | X |  |  |  |  |  |  |
| lVI-rDM | X | X | X |  | X | X | X | X |
| lVI-rBG | X | X |  |  | X | X |  |  |
| rVI-rSM | X | X |  |  |  |  |  |  |
| rSM-rDM | X | X |  |  |  |  |  |  |
| rSM-rBG | X | X |  |  | X | X |  |  |
| rDA-rBG | X | X |  |  |  |  |  |  |
| rDM-rBG | X | X |  |  |  |  |  |  |
| lDA-rDM |  |  | X |  |  |  | X | X |
| Within lDM |  |  |  |  | X | X |  |  |
| lCE-lVI |  |  |  |  | X | X |  |  |
| lDM-lVA |  |  |  |  | X | X |  |  |
| lDM-lDA |  |  |  |  | X | X | X | X |
| lDM-rVA |  |  |  |  | X | X |  |  |
| lDM-rBG |  |  |  |  | X | X |  |  |
| lFP-rVI |  |  |  |  | X | X |  |  |
| lFP-rFP |  |  |  |  | X | X |  |  |
| lFT-rVA |  |  |  |  | X | X |  |  |
| lVA-rDM |  |  |  |  | X | X |  |  |
| lSM-rBG |  |  |  |  | X | X |  |  |
| rVI-rFT |  |  |  |  | X | X |  |  |
| rVI-rDM |  |  |  |  | X | X | X | X |
| Within lFP |  |  |  |  |  |  | X | X |
| lFP-lDA |  |  |  |  |  |  | X | X |
| lVA-lDA |  |  |  |  |  |  | X | X |
| lVA-lVI |  |  |  |  |  |  | X | X |

X denotes significance (p < 0.05) within a statistical approach

Exponentially weighted sliding-window based on Zalesky et al.,^1^ multiplication of temporal derivatives based on Shine et al.^2^

Extent and intensity statistics based on network based statistics^3^

rVI/lVI: right/left visual network*

rSM/lSM: right/left somato-motor network*

rDA/lDA: right/left dorsal attention network*

rVA/lVA: right/left ventral attention network*

rFT/lFT: right/left fronto-temporal network*

rFP/lFP: right/left fronto-parietal network*

rDM/lDM: right/left default mode network*

rHI/lHI: right/left amygdala/hippocampus** (not included in the Power parcellation^4^) rBG/lBG: right/left basal ganglia**

rCE/lCE: right/left cerebellum**

* Networks based on Yeo et al.^5^

** Regions based on Harvard-Oxford atlas

1 Zalesky, A., Fornito, A., Cocchi, L., Gollo, L. L. & Breakspear, M. Time-resolved resting-state brain networks. *Proc Natl Acad Sci U S A* **111**, 10341-10346, doi:10.1073/pnas.1400181111 (2014).

2 Shine, J. M. *et al.* Estimation of dynamic functional connectivity using Multiplication of Temporal Derivatives. *Neuroimage* **122**, 399-407, doi:10.1016/j.neuroimage.2015.07.064 (2015).

3 Zalesky, A., Fornito, A. & Bullmore, E. T. Network-based statistic: identifying differences in brain networks. *Neuroimage* **53**, 1197-1207, doi:10.1016/j.neuroimage.2010.06.041 (2010).

4 Power, J. D. *et al.* Functional network organization of the human brain. *Neuron* **72**, 665-678, doi:10.1016/j.neuron.2011.09.006 (2011).

5 Yeo, B. T. *et al.* The organization of the human cerebral cortex estimated by intrinsic functional connectivity. *J Neurophysiol* **106**, 1125-1165, doi:10.1152/jn.00338.2011 (2011).
